# Accumulation of multiple neurodegenerative disease-related proteins in familial frontotemporal lobar degeneration associated with granulin mutation

**DOI:** 10.1038/s41598-017-01587-6

**Published:** 2017-05-04

**Authors:** Masato Hosokawa, Hiromi Kondo, Geidy E. Serrano, Thomas G. Beach, Andrew C. Robinson, David M. Mann, Haruhiko Akiyama, Masato Hasegawa, Tetsuaki Arai

**Affiliations:** 1 Dementia Research Project, Tokyo Metropolitan Institute of Medical Science, 2-1-6, Kamikitazawa, Setagaya-ku, Tokyo 156-8506 Japan; 2 Histology Center, Tokyo Metropolitan Institute of Medical Science, 2-1-6, Kamikitazawa, Setagaya-ku, Tokyo 156-8506 Japan; 3 Civin Laboratory for Neuropathology, Banner Sun Health Research Institute, 10515 West Santa Fe Drive, Sun City, AZ 85351 USA; 4 Faculty of Biology, Medicine and Health, School of Biological Sciences, Division of Neuroscience & Experimental Psychology, University of Manchester, Clinical Sciences Building, Salford Royal Hospital, Stott Lane, Salford M6 8HD UK; 5 Department of Neuropsychiatry, Division of Clinical Medicine, Faculty of Medicine, University of Tsukuba, 1-1-1, Tennodai, Tsukuba, Ibaraki 305-8576 Japan

## Abstract

In 2006, mutations in the granulin gene were identified in patients with familial Frontotemporal Lobar Degeneration. Granulin transcript haploinsufficiency has been proposed as a disease mechanism that leads to the loss of functional progranulin protein. Granulin mutations were initially found in tau-negative patients, though recent findings indicate that these mutations are associated with other neurodegenerative disorders with tau pathology, including Alzheimer’s disease and corticobasal degeneration. Moreover, a reduction in progranulin in tau transgenic mice is associated with increasing tau accumulation. To investigate the influence of a decline in progranulin protein on other forms of neurodegenerative-related protein accumulation, human granulin mutation cases were investigated by histochemical and biochemical analyses. Results showed a neuronal and glial tau accumulation in granulin mutation cases. Tau staining revealed neuronal pretangle forms and glial tau in both astrocytes and oligodendrocytes. Furthermore, phosphorylated α-synuclein-positive structures were also found in oligodendrocytes and the neuropil. Immunoblot analysis of fresh frozen brain tissues revealed that tau was present in the sarkosyl-insoluble fraction, and composed of three- and four-repeat tau isoforms, resembling Alzheimer’s disease. Our data suggest that progranulin reduction might be the cause of multiple proteinopathies due to the accelerating accumulation of abnormal proteins including TDP-43 proteinopathy, tauopathy and α-synucleinopathy.

## Introduction

Progranulin (PGRN) is a growth factor encoded by a single gene on chromosome 17q21, extremely close to the *MAPT* (tau) gene. PGRN is a 593 amino acid, cysteine-rich protein with a signal peptide and 7.5 highly conserved tandem granulin repeats of a 12-cysteinyl motif. It is involved in the regulation of multiple processes, including neuronal inflammation^1^, wound healing^2, 3^, cell growth^4, 5^, tumorigenesis^6^ and chemoattraction of microglia^7^. Granulin (*GRN*) null mutations were identified in familial frontotemporal dementia (FTD) linked to chromosome 17q21 with tau-negative, ubiquitin-positive inclusions^8, 9^. Many mutations including those due to a frame shift by insertion/deletion or substitution of a nucleotide have been reported, and been shown to generate premature termination codons. *GRN* transcript haploinsufficiency has been proposed as the disease mechanism that leads to the loss of functional PGRN protein. A mutation in the signal peptide may cause mislocalization of PGRN in a protein secretion pathway or induce loss of PGRN function by impairment of its transport^10, 11^. Thus, these mutations are strongly involved in FTD pathogenesis.

Interestingly, loss-of-function *GRN* mutations have been identified in patients clinically diagnosed with Alzheimer’s disease (AD)^12–19^. For example, p.Gly35Arg (c.103G > A)^19^, and a single base pair deletion (c. 154delA) were found in AD, and the latter was shown to cause a frame shift (p.Thr52HisfsX2) creating a premature stop codon^20^. The rs5848 (3′ UTR + 78C > T) variant was also found in AD^21^ and associated with an increased risk of this disease^22^. In addition, *GRN* mutations were found in the accelerating accumulation of abnormal proteins in corticobasal syndrome^10, 23–26^. Furthermore, tau pathology, in addition to TAR-DNA binding protein of 43 kDa (TDP-43) pathology, was found in most members of two families harboring a *GRN* mutation^27^. These findings suggest that a decline in, or dysfunction of, PGRN may cause tau abnormalities, leading to the formation of tau pathology by activation of cyclin-dependent kinases in a P301L tau/*GRN* +/− mouse model^28^. To explore these issues, we performed immunohistochemical staining and biochemical analyses on human familial *GRN* mutation cases and examined whether *GRN* reduction accelerates the accumulation of neurodegenerative-related proteins other than TDP-43.

In this study, using a novel, highly sensitive immunohistochemical method employing free-floating sections, we noted massive phosphorylated-tau-positive staining in some familial *GRN* mutation cases. Notably, in these same cases, we also observed significant phosphorylated α-synuclein positive staining. Additionally, detergent-insoluble tau and α-synuclein proteins were detected by immunoblot analysis. Similar tau pathology was not seen in other *GRN* mutation cases when employing standard immunohistochemistry based on paraffin-embedded sections. Our results suggest that at least some cases with *GRN* mutations may show a hitherto unrecognized accelerated pathological accumulation of tau and α-synuclein.

## Materials and Methods

### Ethics Statement

All patients, or in some cases in which the patient had died, next of kin, provided written consent for autopsy and postmortem analyses for research purposes. Written informed consent was obtained from all patients. This study was approved by the Ethics Committee of the Tokyo Metropolitan Institute of Medical Science (permission No. 15-1 and 15-5(1)), the Banner Sun Health Research Institute and University of Manchester. The study was performed in accordance with the ethical standards laid down in the 1964 declaration of Helsinki and its later amendments.

### Cases

The brain tissues used in Study A from four patients with *GRN* and three controls were from the Banner Sun Health Research Institute (Sun City, AZ), Brain and Body Donation Program^29, 30^. The additional nine *GRN* mutation cases in Study B were from the Manchester Brain Bank (UK). Ten control cases in Study B were registered in the autopsy archives of Dementia Research Project, Tokyo Metropolitan Institute of Medical Science. Case details are presented in Table 1. Seven different *GRN* mutations were recorded. Briefly, Case 1 had a c.1252C > T mutation resulting in p.Arg418X. Cases 2, 4, 9 and 12 had a c.1477C > T mutation resulting in p.Arg493X. A point mutation in a translation initiation codon (c.1A > C) predicted reduced mRNA levels in case 3. Three patients (cases 8, 14 and 16) shared c.1355delG mutation resulting in p.V452WfsX38. Case 10 had a c.1402C > T mutation resulting in p.Q468X and case 13 had a c.90_91insCTGC mutation resulting in p.C31LfsX34. Two patients (cases 11 and 15) shared c.388_391delCAGT mutation resulting in p.Q130SfsX124.

**Table 1:** Description of the *GRN* mutation and control cases used in the initial study (Study A, No. 1–7) and the additional cases used in the second study (Study B, No. 8–26).

Case No.	Gender	Age at death	Clinical diagnosis	Pathological diagnosis	Mutation (cDNA)	Mutation (Protein)	APOE	Tau haplotype	Brain weight (g)
1	M	54	AD/PiD	FTLD-TDP Type A/HS	c.1252C > T	p.R418X	n.d.	H1/H2	980
2	F	56	FTD/AD	FTLD-TDP Type A/LBD	c.1477C > T	p.R493X	n.d.	n.d.	940
3	F	72	AD	FTLD-TDP Type A/AD	c.1A > C	p.0	n.d.	H1/H1	1120
4	M	55	AD	FTLD-TDP Type A/HS/LBD	c.1477C > T	p.R493X	n.d.	H1/H1	800
5	M	73	Myeloid leukemia	Age changes only	None	None	n.d.	n.d.	1240
6	M	76	Multiple myeloma				n.d.	n.d.	1375
7	M	79	Prostate cancer				n.d.	n.d.	1428
8	F	71	FTD	FTLD-TDP Type A	c.1355delG	p.V452WfsX38	E3/E3	H2/H2	955
9	F	61	FTD		c.1477C > T	p.R493X	n.d.	n.d.	900
10	F	66	FTD		c.1402C > T	p.Q468X	E3/E3	H1/H1	1100
11	F	71	PNFA		c.388_391delCAGT	p.Q130Sfs124	E3/E3	H1/H1	863
12	M	66	FTD		c.1477C > T	p.R493X	E3/E3	H2/H2	1495
13	M	73	Prog Anomia		c.90_91insCTGC	p.C31LfsX34	E3/E3	n.d.	1250
14	M	71	CBD/PAX		c.1355delG	p.V452WfsX38	E3/E4	H2/H2	925
15	M	73	PiD		c.388_391delCAGT	p.Q130Sfs124	E3/E3	n.d.	980
16	M	72	PNFA		c.1355delG	p.V452WfsX38	E3/E4	H1/H2	870
17	M	66	Narcolepsy	Age changes only	None	None	n.d.	n.d.	1221
18	F	63	Spinal muscular atrophy				n.d.	n.d.	1221
19	M	51	Sch				n.d.	n.d.	1332
20	M	50	Candidal meningitis				n.d.	n.d.	1300
21	F	53	Atypical psychosis				n.d.	n.d.	?
22	M	63	Sch/Parkinson’s syndrome				n.d.	n.d.	?
23	M	51	Sch/Aspiration pneumonia				n.d.	n.d.	1494
24	M	60	Sch/Aspiration pneumonia				n.d.	n.d.	1435
25	M	63	Sch/Pseudomonas aeruginosa pneumonia				n.d.	n.d.	1430
26	M	57	Sch/Multiple myeloma				n.d.	n.d.	1149

n.d.: not determined, Gender: F, female; M, Male.

AD, Alzheimer’s disease; CBD, corticobasal degeneration; FTD, frontotemporal dementia; HS, hippocampal sclerosis; LBD, Lewy body disease; PAX, apraxia;

PiD, Pick’s disease; PNFA, progressive non-fluent aphasia; Prog Anomia, progressive anomia; Sch, schizophrenia.

### Histochemical analysis

Study A: For immunohistochemistry, sections fixed in 4% paraformaldehyde and preserved in 20% sucrose were cut serially on a freezing microtome at 40 μm thickness, collected in maintenance solution, and immunostained as free-floating sections (cases 1–7). Sections were incubated with 1% H_2_O_2_ for 30 min to eliminate endogenous peroxidase activity and were pretreated by autoclaving for 10 min in 10 mM sodium citrate buffer, pH6.0, at 121 °C. Sections were incubated for 24 hours with AT8 (1:1,000, Innogenetics, Ghent, Belgium), anti-TDP-43-pS409/410 antibody (1:1,000, Dr. Hasegawa), anti-phosphorylated α-synuclein antibody (1175, 1:1,000, Dr. Akiyama), E50 (for amyloid β, 1:10,000, Dr. Akiyama), RD3 (1:1,000, Merck Millipore), anti-4R tau (1:1,000, Dr. Hasegawa) or anti-FUS (1:1000, Sigma-Aldrich, St. Louis, MO, USA).

Study B: As we could not obtain sections in cases 8–26 that had been fixed and preserved under the same conditions as cases 1–7, we used formalin-fixed, paraffin-embedded sections instead. Therefore, sections from cases 8–26 were cut at 10 μm thickness, deparaffinized, incubated with 1% H_2_O_2_ for 30 min to eliminate endogenous peroxidase activity in the tissue, then pretreated for 10 min in 10 mM sodium citrate buffer, pH6.0 at 110 °C. Sections were then treated with formic acid for 10 min (for α-synuclein staining) or 30 min (for tau staining). For tau immunostaining, sections were incubated in 10 μg/ml of trypsin (Sigma-Aldrich) at 37 °C for 10 min. They were also incubated with AT8 and anti-phosphorylated α-synuclein antibody (1175), overnight, as in Study A. Antibody labeling was performed by incubation with goat anti-rabbit IgG (1:1,000, Vector Laboratories, Burlingame, CA, USA) or horse anti-mouse IgG (1:1,000, Vector Laboratories) for 3 hours. The antibody labeling was visualized by incubation with avidin-biotinylated horseradish peroxidase complex (ABC Elite, Vector Laboratories, 1:1,000) for 3 hours, followed by incubation with a solution containing 0.01% 3,3′-diaminobenzidine, 1% nickel ammonium sulfate, 0.05 M imidazole and 0.00015% H_2_O_2_ in 0.05 M Tris-HCl buffer, pH 7.6. Counter nuclear staining was performed with Kernechtrot stain solution (Merck, Darmstadt, Germany) or hematoxylin (Muto Pure Chemicals, Tokyo, Japan). The sections were then rinsed with distilled water, mounted on glass slides, treated with xylene, and coverslipped with Entellan (Merck). Tissue sections (cases 1–4) were also stained using a modified Gallyas-Braak method. Photographs were taken with a BX53 microscope (Olympus, Tokyo, Japan).

For fluorescent immunohistochemistry, free floating sections were incubated for 10 min with 0.1% Sudan black/70% ethanol solution and then incubated for 24 hours with AT8 (1:500), anti-TDP-43-pS409/410 antibody (1:500), anti-phosphorylated α-synuclein antibody (1175, 1:500) or pSyn#64 (1:500, Wako, Osaka, Japan). Antibody labeling was visualized by incubation with Alexa 488- or 568-labeled anti-rabbit IgG (1:100, Invitrogen, Carlsbad, CA, USA) or Alexa 488- or 568-labeled anti-mouse IgG (1:100, Invitrogen) for 2 hours. The sections were coverslipped with ProLong Gold with 4′,6-diamidino-2-phenylindole (Invitrogen). Photographs were taken with a BZ-8000 (Keyence, Osaka, Japan).

### Sequential fractionation of brain extracts

Frozen brain samples (middle frontal gyrus, approximately, 0.2 g) were homogenized in 10 volumes of A68 buffer (10 mM Tris-HCl, pH 7.5, 0.8 M NaCl, 1 mM ethylene glycol bis-N, N, N′, N′-tetraacetic acid, 10% sucrose) containing 1% sarkosyl followed by incubation for 30 min at 37 °C. Each brain homogenate was centrifuged at 15,000 rpm for 10 min at 4 °C, and the supernatant was collected. The supernatant was then centrifuged at 136,000× g for 20 min at 4 °C. The sarkosyl-insoluble pellet was sonicated in 60 μl of SDS-PAGE sample buffer containing 4 M urea.

### Histopathological assessments

Age-related plaque scores were determined using the Braak staging^31^. For purpose of this protocol, the letter corresponds to the following assessment: 0 = No Aβ deposits, A = initial Aβ deposits can be found in basal portions of the isocortex, B = Aβ deposits can be shown in virtually all isocortical association areas, C = Aβ deposits can be seen in all areas of the isocortex, including the sensory and motor core fields. For the evaluation of neurofibrillary changes (tau deposition), Braak staging was applied^31^. In this protocol, the number corresponds to the following assessment of the area of tau deposition: Stage I = transentorhinal cortex, Stage II = entorhinal cortex, Stage III = hippocampus-subiculum, Stage IV = temporal cortex, Stage V = parietal cortex, and Stage VI = occipital cortex. The degree of accumulation of tau and α-synuclein was also evaluated qualitatively and a score ranging from – (negative) to +++ (severe) was assigned.

### Immunoblotting analyses

For immunoblotting, brain extracts from the *GRN* mutation cases were boiled for 5 minutes with SDS-PAGE sample buffer (60 mM Tris-HCl, pH 6.8, containing 2% SDS, 10% glycerol, 0.025% bromophenol blue and 5% mercaptoethanol) and loaded onto a 5–20% acrylamide minigel. Loaded samples were electrophoresed for 75 minutes at 200 V with molecular weight markers (Bio-Rad, Hercules, CA, USA). Electrophoresed proteins were transferred onto a polyvinylidene difluoride membrane (Merck Millipore) and subjected to 200 mA for 60 minutes. The printed membranes were blocked with 3% gelatin for 15 min and then incubated in a primary antibody solution, T46, (1:1,000, Innogenetics), RD3 (1:1,000, Merck Millipore), anti-4R tau (1:1,000, Dr. Hasegawa) for overnight at room temperature. Following incubation with the secondary anti-mouse or anti-rabbit antibody (1:50,000, Bio-Rad), immunoreactivity was detected by the chemiluminescence method using a Super Signal West Dura Extended Duration Substrate (Thermo Fisher Scientific, West Palm Beach, FL, USA) and was visualized with a LAS-4000 mini (GE Healthcare UK Ltd., Buckinghamshire, UK). For α-synuclein immunoblot, the printed membranes were incubated in a primary antibody solution, anti-phosphorylated α-synuclein antibody (1175, 1:1,000) or pSyn#64 (1:1,000) for overnight at room temperature. Following incubation with the biotinylated-secondary anti-rabbit or anti-mouse antibody (1:500, Vector Laboratories), followed by Vectastain Elite ABC kit (Vector Laboratories). Immunoreactivity was detected by 3,3′-diaminobenzidine with nickel chloride.

## Results

### *GRN* mutation cases used in the present study

The age, gender, clinical and pathological diagnoses and genetic information on the familial *GRN* mutation cases and control cases used in this study is summarized in Table 1. None of the *GRN* mutation cases examined in this study had *MAPT* mutation, and the *MAPT* haplotype was determined to be H1/H1 in cases 3, 4, 10 and 11, H1/H2 in cases 1 and 16, H2/H2 in cases 8, 12 and 14 (Table 1). The *MAPT* haplotype of cases 2, 9, 13 and 15 were unknown.

### TDP-43 accumulation in *GRN* mutation cases

**Figure 1:**
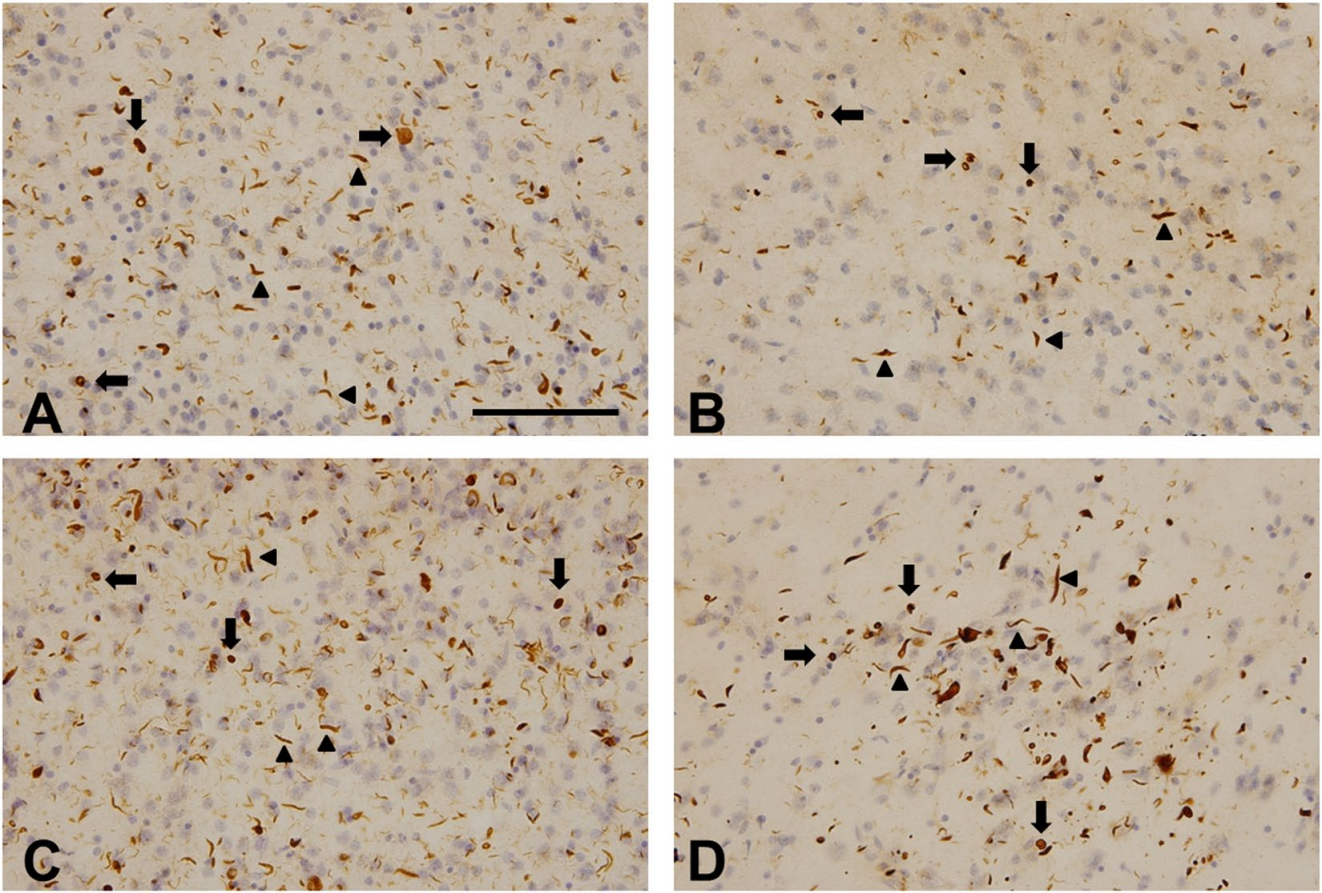
Immunohistochemical staining of the temporal lobe of *GRN* mutation cases with antibody to phosphorylated TDP-43. Numerous neuronal cytoplasmic inclusions (arrows) and dystrophic neurites (arrowheads) were stained with anti-TDP-43-pS409/410 antibody in cases 1 (**A**), 2 (**B**), 3 (**C**) and 4 (**D**). The sections were counterstained with hematoxylin. The scale bar in (**A**) applies to all photomicrographs (100 μm).

Phosphorylated TDP-43 inclusions were visualized by anti-TDP-43-pS409/410 antibody. Neuronal cytoplasmic inclusions (NCIs) and dystrophic neurites (DNs) were observed in the cerebral cortices of *GRN* mutation cases 1–4 (Fig. 1A–D) and cases 8–16 (data not shown). All thirteen *GRN* mutation cases were categorized as “Type A” according to the classification system of Mackenzie *et al*.^32^, consistent with that type of TDP-43 pathology previously reported in *GRN* mutation cases (Tables 2 and 3). All control cases (5–7 and 17–26) were negative for phosphorylated TDP-43 (Tables 2 and 3).

**Table 2:** Summary of immunohistochemical analyses of the initial study (Study A, No. 1–7).

Case No.	Case	Age	TDP-43	Aβ	Tau	Tau (neuronal/glial)				α-syn		
						Hip-Ent	Amygdala	Temporal	Frontal	Hip-Ent	Amygdala	Temporal
1	*GRN* mutation	54	FTLD-TDP (Type A)	A	IV	+++/+	++/+	+/+	+/+	++	+	+
2		56		0	IV	+++/++	+++/++	+++/++	++/++	+	N.A.	+
3		72		C	V	+++/+	++/+	+++/++	+++/-	N.A.	+	+
4		55		A	IV	+++/+	+/+	+/+	+/+	+++	+	++
5	Control	73	Negative	B	IV	++/−	++/+	++/+	N.A.	+	+	−
6		76		A	I	+/−	+/−	+/−	N.A.	+	+	−
7		79		A	I	++/−	+/+	+/+	N.A.	+	+	−

+mild, ++moderate, +++severe, −negative, N.A.: not available.

**Table 3:** Summary of immunohistochemical analyses of the second study (Study B, No. 8–26).

Case No.	Case	Age	TDP-43	Aβ	Tau	α-syn
8	*GRN* mutation	71	FTLD-TDP (Type A)	A	I	Negative
9		61		0	IV	
10		66		0	II	
11		71		0	II	
12		66		0	I	
13		73		0	II	
14		71		B	I	
15		73		0	0	
16		72		A	II	
17	Control	66	Negative	0	II	
18		63		0	0	
19		51		0	I	
20		50		A	0	
21		53		0	I	
22		63		0	I	
23		51		0	0	
24		60		A	I	
25		63		0	0	
26		57		0	0	

### Tau accumulation in *GRN* mutation cases

**Figure 2:**
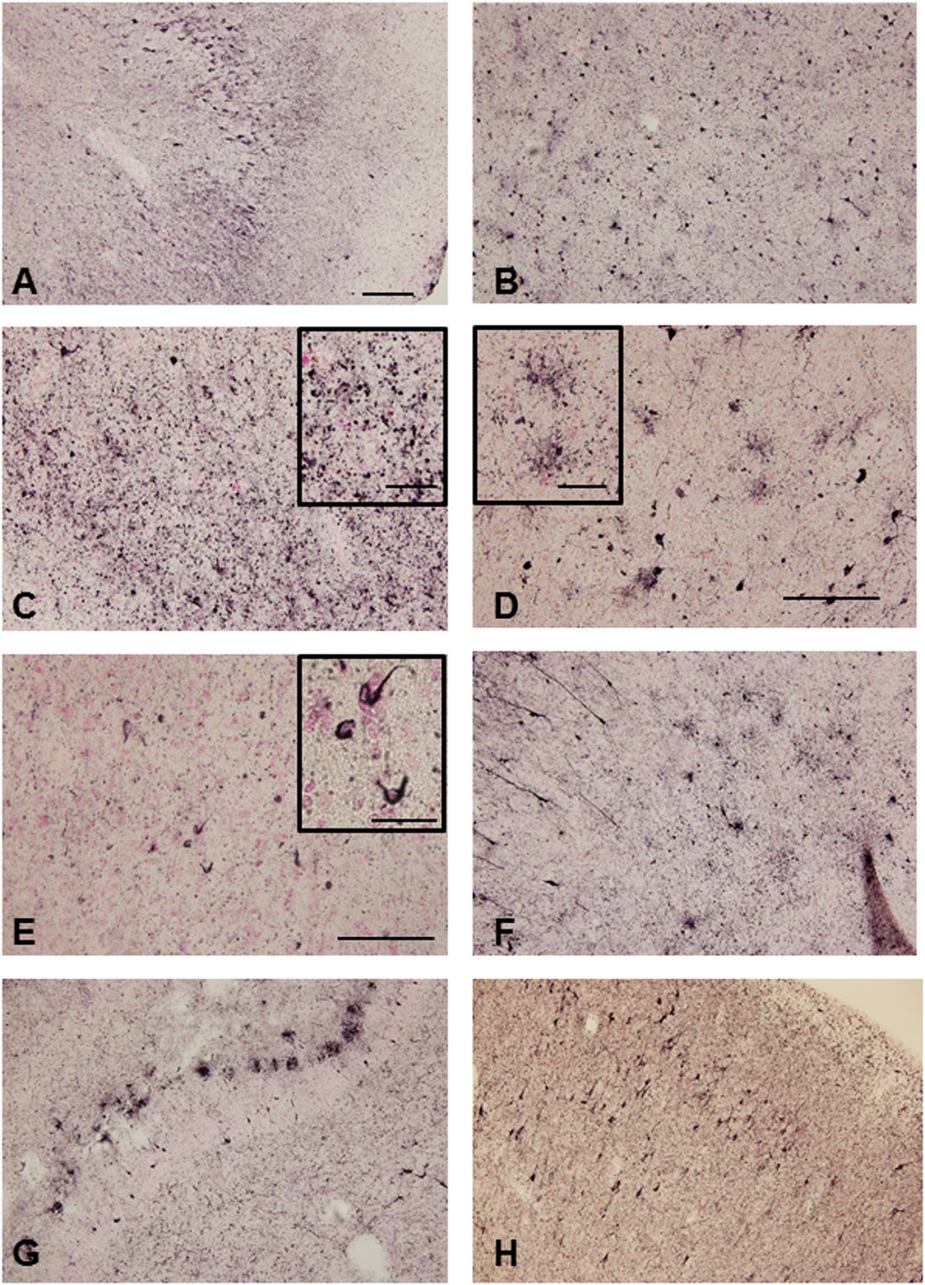
Photomicrograph of phosphorylated tau immunohistochemistry of the temporal lobe of the *GRN* mutation cases. Massive AT8-positive structures were observed in the hippocampus (**A**), amygdala (**B**), inferior temporal cortex (**C**) in case 2. AT8-positive astrocytes were observed in the temporal cortex (**D**) and AT8-positive oligodendrocytes in the white matter of temporal lobe (**E**) in case 2. AT8-positive deposition was also detected in amygdala in case 1 (**F**), hippocampus in case 3 (**G**) and entorhinal cortex in case 4 (**H**). The sections were counterstained with Kernechtrot stain solution. The scale bar in A applies to (**B**,**C**,**D**,**F**,**G** and **H**) (200 μm), in E (100 μm), respectively. The scale bars in the inserts are 50 μm (**C** and **D**) and 25 μm (**E**), respectively.

We observed a considerable number of tau-positive neurons, astrocytes and oligodendrocytes in all 4 Study A *GRN* mutation cases by either AT8 immunostaining (Fig. 2, and Table 2) or Gallyas silver staining (data not shown). In particular, case 2 showed massive AT8-positive structures in the entorhinal cortex, hippocampus (Fig. 2A), amygdala (Fig. 2B), temporal cortex (Fig. 2C), insula. In the temporal lobe, the majority of tau-positive neuronal cytoplasmic staining appeared as pretangle-like forms (Fig. 2A,F,G,H). In the neuropil, fine tau–positive granules were abundant (Fig. 2C). The size of most of these granules appeared smaller than the tau-positive grains observed in argyrophilic grain disease (AGD) brains, and they were negative for Gallyas-silver staining (data not shown).

Furthermore, tau-positive astrocytic structures, resembling “bush-like” astrocytes previously reported in AGD^33^, were found in the cortex in all cases in Study A. Their morphology was significantly different from the tufted-astrocytes in progressive non-fluent aphasia (PSP) patients, the astrocytic plaques in corticobasal degeneration (CBD) or the ramified astrocytes in Pick’s disease (Fig. 2D). In the white matter, tau-positive oligodendroglial coiled bodies were observed (Fig. 2E).

Gallyas silver staining also revealed structures similar to those stained with AT8, including neurofibrillary tangles (NFTs), threads, and astrocytic and oligodendrocytic structures (data not shown). In the hippocampal region, many NFTs were found in cases 2 and 4 by both AT8 immunostaining and Gallyas silver staining (data not shown). The control cases of Study A showed mild to moderate AT8-positive structures and less glial tau deposition compared to *GRN* mutation cases (Table 2). Case 1, 2, 4 and 5 exhibited tau deposition that corresponded to Braak stage IV, Case 3 corresponded to Braak stage V and Case 6 and 7 corresponded to Braak stage I, respectively (Table 2). The degree of accumulation of tau was evaluated qualitatively and a score ranging from – (negative) to +++ (severe) was assigned (Supplementary Figure 1 and Table 2).

In Study B, eight of nine *GRN* mutation cases exhibited some AT8 immunoreactivity (Supplementary Figure 2 and Table 3), but the levels of phosphorylated tau deposition were up to Braak stage II except for Case 9 (Table 3), dissimilar to that seen in *GRN* mutation cases of Study A. No tau deposition or only Braak stage I-II were observed in the control cases of study B (Table 3). The tau pathology in the *GRN* mutation cases (Study A) was also detected by 3-repeat (3R)-tau (RD3) and 4-repeat (4R)-tau (anti-4R) specific antibodies indicating that both 3R and 4R tau accumulation was present (Data not shown).

### α-synuclein accumulation in *GRN* mutation cases

**Figure 3:**
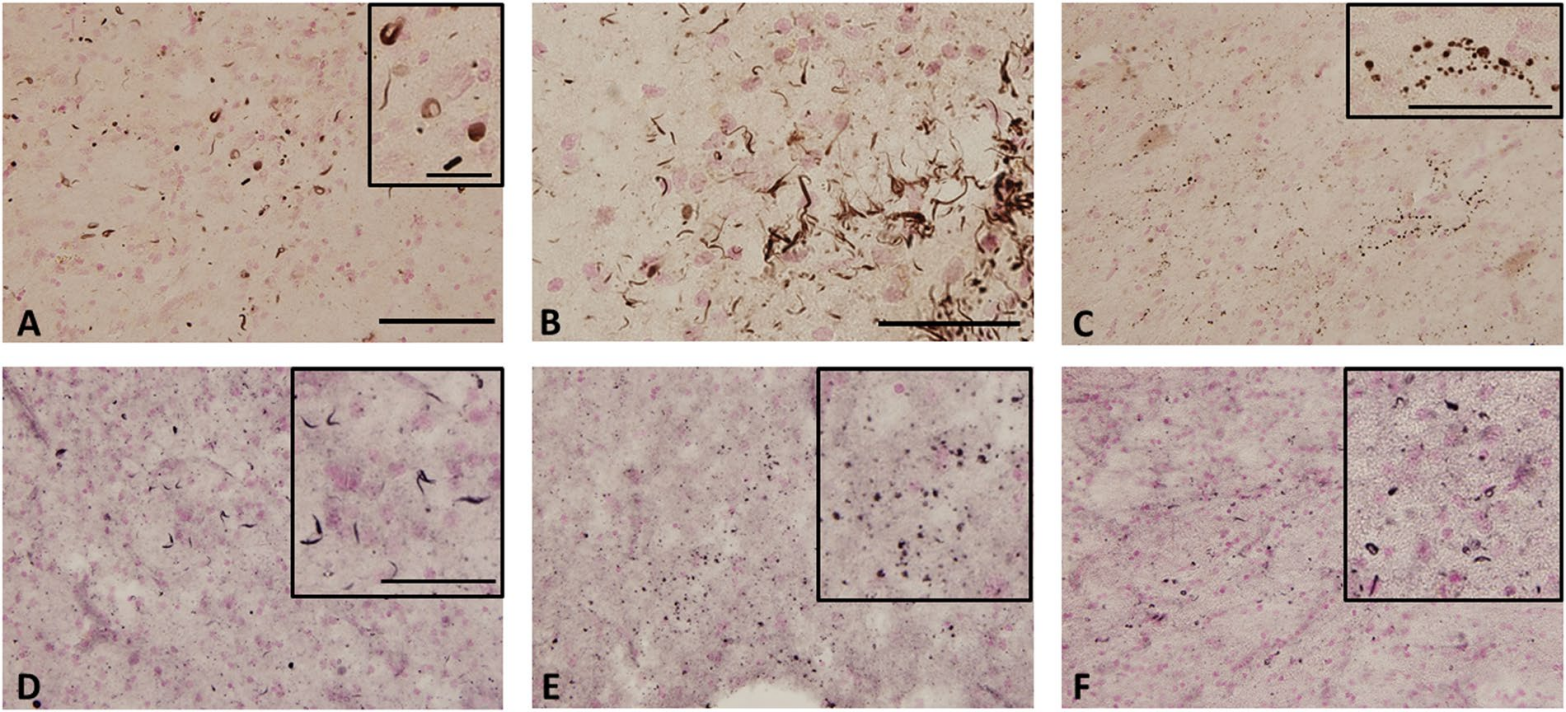
Immunohistochemical staining of phosphorylated α-synuclein in the temporal lobe of the *GRN* mutation cases. Phosphorylated α-synuclein -positive structures were observed in the inferior temporal cortex (**A**), superior temporal cortex (**B**) and tuberomammillary nucleus (**C**) in case 4, and in the temporal cortex in case 1 (**D**), case 2 (**E**) and case 3 (**F**). Sections were counterstained with Kernechtrot stain solution. The scale bar in (**A**) applies to (**C**–**F**) (200 μm). The scale bars in (**B**), in insert (**A**,**C** and **D**–**F**) are 50 μm.

Immunohistochemistry using an antibody to phosphorylated α-synuclein, revealed small round or dot-like structures and short thread-like structures in the temporal lobe (Fig. 3A–F), and oligodendroglial coiled body-like structures in the temporal white matter (data not shown) in cases 1–4 of Study A. The degree of accumulation of α-synuclein was evaluated qualitatively and a score ranging from – (negative) to +++ (severe) was assigned. In particular, case 4 exhibited atypical α-synuclein deposition in the temporal cortex (Fig. 3A,B and Table 2). Phosphorylated α-synuclein positive structures were not found in Study B using paraffin-embedded sections of *GRN* mutation cases (cases 8–16, Table 3) and control cases (cases 17–26, Table 3).

### Amyloid β deposition in *GRN* mutation cases

**Figure 4:**
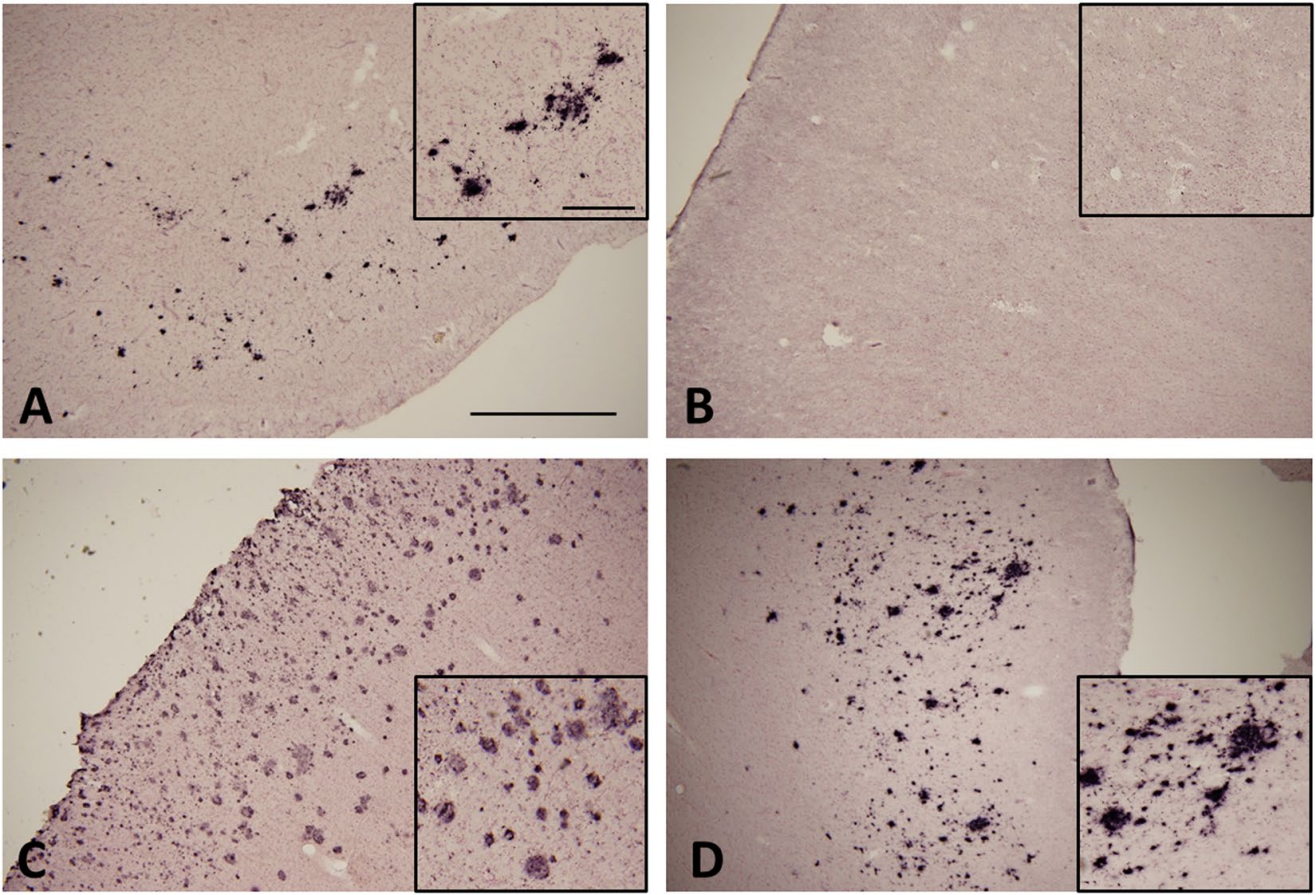
Immunohistochemical staining of amyloid β in the temporal lobe of *GRN* mutation cases. Amyloid β (Aβ) was observed in cases 1 (**A**), 3 (**C**) and 4 (**D**), but not in case 2 (**B**). Most Aβ-positive structures consisted of diffuse plaques. The scale bar in (**A**) applies to all photomicrographs (1.0 mm) and the scale bar in insert A applies to insert (**B**–**D**) (250 μm).

Aβ deposition was found in the temporal lobe in three of four *GRN* mutation cases in Study A (Fig. 4 and Table 2). In the cases 1 and 4, Aβ pathology was present mostly as diffuse plaques, corresponding to Braak stage A (Fig. 4A and D). In case 2, there was no Aβ pathology (Fig. 4B) but case 3 corresponded with Braak stage C (Fig. 4C). Of the control cases in Study A, three were similar to Braak stage A, but one (case 5) corresponded to Braak stage B. In Study B, Aβ accumulation in almost all cases corresponded to Braak stage 0, the others showing Braak stage A (Table 3).

### Immunoblot analyses

**Figure 5:**
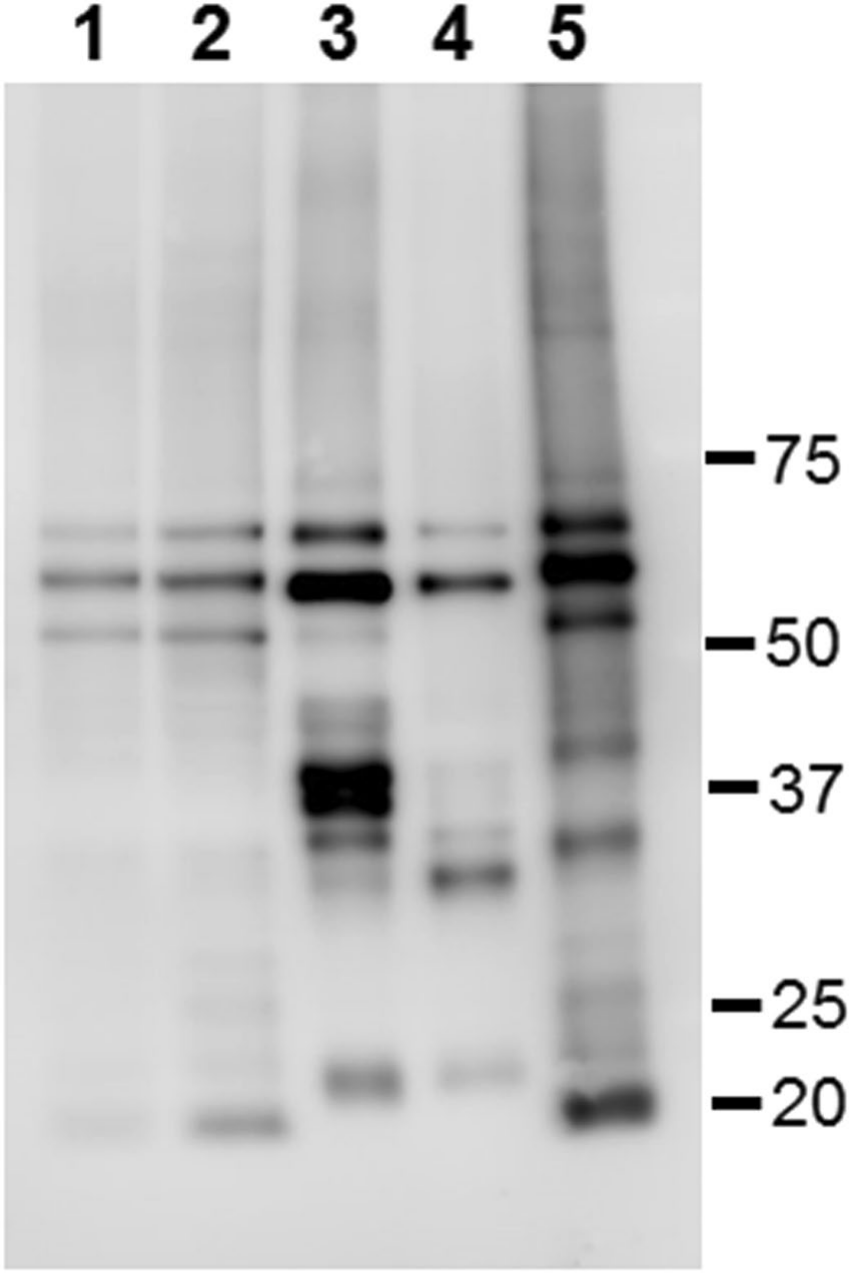
Comparison of the banding patterns of sarkosyl-insoluble tau on immunoblotting between cases with a *GRN* mutation and those with other tauopathies. Immunoblotting analysis was visualized using the T46 antibody for detecting tau in the sarkosyl-insoluble fraction from cases 3 (lane 1) and 4 (lane 2) with *GRN* mutations, and a case each of CBD (lane 3), PSP (lane 4) and AD (lane 5). Molecular weight markers are shown on the right (kDa).

Biochemical features of accumulated tau in cases of *GRN* mutation (Fig. 5, Case 3: lane 1, Case 4: lane 2) were compared with those of other tauopathies including CBD (lane 3), PSP (lane 4) and AD (lane 5) by immunoblot analysis of the sarkosyl-insoluble fraction using C-terminal tau antibody (T46) (Fig. 5). The major tau band pattern in *GRN* mutation cases was triplets of 68, 64 and 60 kDa, similar to that in AD, but different from that in CBD and PSP (Fig. 5). *GRN* mutation cases were also detected by 3R-tau (RD3) and 4R-tau (anti-4R) specific antibodies indicating both 3R and 4R tau accumulation (Supplementary Figure 3). *GRN* mutation cases were also studied with anti-phosphorylated α-synuclein antibodies (1175 and pSyn#64) for cases 1–4. Very faint bands of phosphorylated α-synuclein were observed at 16 kDa. (Supplementary Figure 4).

### Fluorescence immunohistochemistry

**Figure 6:**
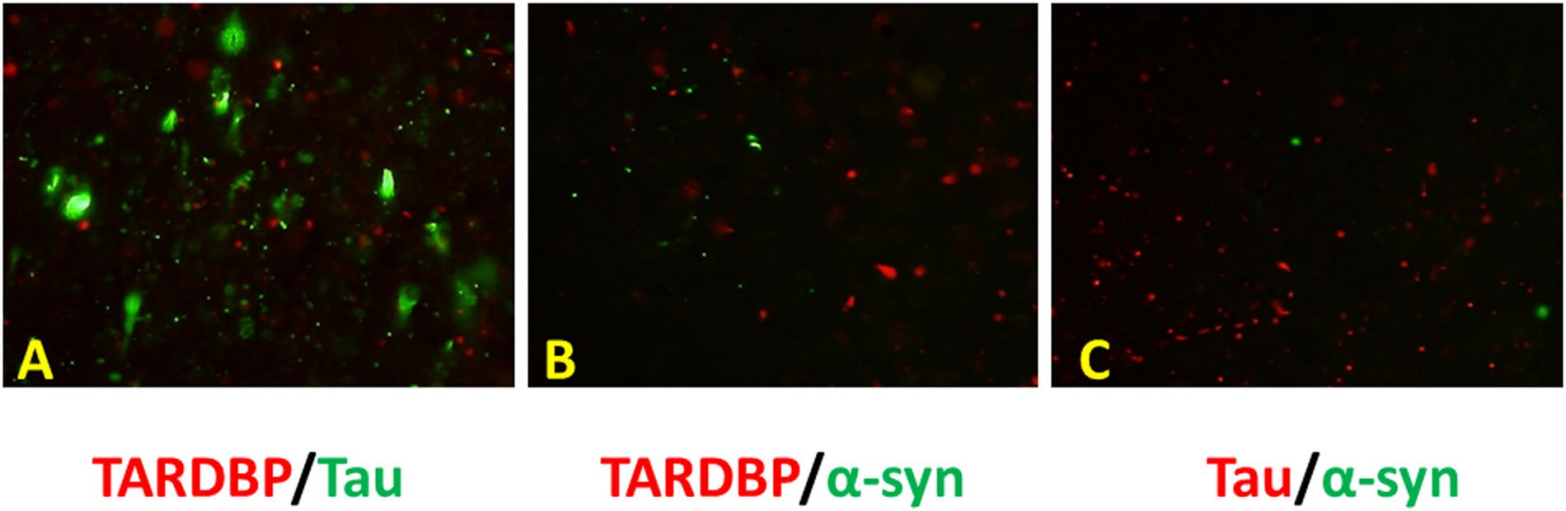
Immunofluorescent double-staining of accumulated proteins in the temporal lobe in *GRN* mutation case. Phosphorylated TDP-43, α-synuclein and tau immunoreactivity were obserbed in the temporal lobe of *GRN* mutation case 4. pTDP-43 (red)/pTau (green) (**A**), pTDP-43 (red)/pα-synuclein (green) (**B**) and pTau (red)/pα-synuclein (green) (**C**).

Fluorescent double-staining of the temporal lobe of the *GRN* mutation case 4 was performed to examine whether TDP-43/tau (Fig. 6A), TDP-43/α-synuclein (Fig. 6B) or tau/α-synuclein (Fig. 6C) were co-localized in the abnormal structures. Colocalization of these proteins was very infrequent in most abnormal structures.

## Discussion

The results of the present study show that *GRN* mutations causing PGRN reduction may accelerate the intracellular accumulation of not only TDP-43 but also tau and α-synuclein in the brains of familial FTD patients with *GRN* mutations. This suggests that *GRN* mutations causing PGRN reduction may be causative or represent risk factors for multiple proteinopathies (TDP-43 proteinopathy, tauopathy or α-synucleinopathy).

Immunohistochemical analyses of phosphorylated TDP-43 revealed a considerable number of neuronal cytoplasmic inclusions and dystrophic neurites in all *GRN* mutation cases (Fig. 1, Tables 2 and 3). In FTLD-TDP, TDP-43 pathology falls within four histological subtypes (types A-D) based on the predominant type of TDP-43-positive structures exhibited^32^. Type A is characterized by numerous short dystrophic neurites and crescentic or oval neuronal cytoplasmic inclusions. Cases of FTLD-TDP with a *GRN* mutation invariably display type A pathology^34–36^, and present observations were in accordance with this.

The very high sensitivity staining method that we performed for Study A (cases 1–7) revealed Case 2 to show atypical tauopathy with massive tau deposition in neuron, astrocytes and oligodendrocytes without Aβ deposition. Case 4 was atypical synucleinopathy with diffuse α-synuclein positive structures that were observed mainly in the neocortex. Case 3 exhibited massive Aβ deposition corresponding to Braak Stage C and tau deposition corresponding to Braak stage V in AD pathology. Case 1 exhibited tau deposition that corresponded to Braak stage IV. It is possible that case 3 might be an incidental complication of AD because the age was late 70 s’. The pathology in the other two cases (case 2 and 4), however, is very rarely observed in the normal aging brain at mid-50 years of age. The control cases in Study A also had levels of tau deposition that corresponded to Braak stage I to IV (Table 2), but the average age was higher than that of the *GRN* mutation cases. We compared abnormal tau deposition using the paraffin-embedded tissues of *GRN* mutation and control cases, and there were significantly differences (Study B, Table 3). Though Study B was less obvious differences than Study A. Braak *et al*. reported that for Braak NFT stage III-IV, the ratio was less than 10% at ages 50 s’ to 60 s’^37^, so that our *GRN* mutation cases in Study A showed tau accumulation atypical for normal aging.

It has been widely accepted for the past decade that there is no tau deposition in *GRN* mutation brains. However, using high-sensitivity immunohistochemical staining, we have found that hyper-accumulated tau and α-synuclein can occur in younger *GRN* mutation cases. Part B of the present study, using paraffin-embedded tissues, however, showed only mild tau deposition, as has been previously reported^8, 9^. Hence, *GRN* mutation may accelerate deposition of tau and α-synuclein but the level of abnormal protein deposition seen in routine paraffin-embedded sections from *GRN* cases might not be as strong as that seen than in free-floating sections and therefore go unrecognized. Re-analysis might be necessary in other *GRN* mutation cases using this high-sensitivity immunohistochemical staining method, or immunoblot analyses on frozen brain tissue, in order to gain a fuller appreciation of the level of tau pathology present in such cases.

Our previous report made mention of the fact that a *GRN* mutation in P301L tau transgenic mice affected phosphorylated tau deposition^28^. The results of the present study support our previous observations in mice. It has been reported that PGRN deficiency causes lysosomal dysfunction^38^. We hypothesized that lysosomal dysfunction might reduce protein degradation in brain cells allowing aggregation-prone neurodegenerative disease-related proteins to deposit more easily.

The features of tau pathology in *GRN* mutation cases in this study are of predominantly neuronal pretangles, abundant fine granules in the neuropil, and astrocytic and oligodendroglial pathology. It is interesting that fine tau-positive granules were reported in the striatum of a brain with a *GRN* c.709-2A > G mutation^27^. Among tauopathies, the tau pathology most similar to our cases might be found in AGD. However, the size of the fine granules in our cases seemed smaller than that of the grains in AGD and they were negative for Gallyas-silver staining (data not shown). Although the form of tau-positive astrocytes in our cases was similar to the “bush-like” astrocytes in AGD, their Gallyas-positive status in contrast to the Gallyas-negative status of the “bush-like” AGD astrocytes^33^. No FUS accumulation was found in any *GRN* mutation cases in Study A, thus there might be no or little relationship between the *GRN* mutation and FUS deposition (data not shown).

Immunoblot analysis of our cases using C-terminal tau antibody revealed that the banding patterns of the full-length tau in the sarkosyl-insoluble fraction appeared to be essentially the same as that seen in AD (Fig. 5). The staining using three and four repeat tau specific antibodies revealed that tau in the sarkosyl-insoluble fraction consists of both forms of tau (Supplementary Figure 2). These results suggest that accumulated tau in cases of *GRN* mutation cases contains six tau isoforms just as in AD. However, the distribution of fine granular tau and the lack of any or only light Aβ accumulation (Fig. 4) is different from AD pathology. Tau isoforms in *GRN* mutation cases were biochemically different from those in CBD and PSP (Fig. 5). Cases of *GRN* mutation may therefore represent a different tauopathy from that of AD, CBD, PSP and AGD.

In addition to neuronal and glial tau accumulation, the present study also revealed α-synuclein-positive structures, including small round, dot-like or thread-like structures in the cortex and oligodendroglial coiled body-like structures in the white matter in the *GRN* mutation cases in Study A (cases 1–4, Fig. 3). Case number 4 showed particularly striking phosphorylated α-synuclein pathology. However, the nine paraffinized *GRN* mutation cases showed no α-synuclein-positive structures. This discrepancy might be caused by fixation or preservation methods. Leverenz and colleagues reported that α-synuclein pathology was observed in two of seven brains with a familial *GRN* mutation. One case showed brainstem α-synuclein pathology while the other was cortical^27^.

Accumulations of phosphorylated-tau, α-synuclein and TDP-43 were reported in the brains of Guam/Kii-amyotrophic lateral sclerosis (ALS)-parkinsonism-dementia complex (PDC) patients^39^. The triplet tau band patterns (68, 64, and 60 kDa) of immunoblot analysis in the sarkosyl-insoluble fraction of *GRN* mutation cases (Fig. 5) appeared to be essentially the same among cases with the *GRN* mutation, AD and Guam/Kii ALS-PDC. Fine tau-positive granules were also reported in the cerebral white matter of Guam-PDC cases, in which the morphology seemed to resemble that of our cases. Hazy astrocytes were observed in Guam-PDC cases, but their morphology seemed to differ from that of our cases. To date, no *GRN* mutations in Guam/Kii ALS-PDC cases have been reported. Very recently we reported that the *GRN* mutation leads lysosomal dysfunction^40^. We speculated that there could be common pathway(s) in lysosomal function or that these diseases have something common features of protein aggregation because of the similarities (TDP-43, tau, α-synuclein deposition and 3R/4R tau isoform aggregation).

In conclusion, when using a highly sensitive free-floating immunohistochemical technique combined with western blotting, we have shown widespread pathological tau and α-synuclein deposition in neurons and glial cells in familial *GRN* mutation cases that are not apparent when using standard immunohistochemical methods based on routine paraffin embedded sections. Although, the number of samples for this study was small and we recognize the limits of this study, our findings suggest that the pathologies seen in *GRN* mutation cases may possibly be renamed “neuronoglial multiple proteinopathies”.

## Electronic supplementary material

Supplementary Information
