# Supplementary material for: Accumulation of multiple neurodegenerative disease-related proteins in familial frontotemporal lobar degeneration associated with granulin mutation
Source: Sci Rep. 2017 May 4;7:1513. doi: 10.1038/s41598-017-01587-6 (PMC5431430; doi:10.1038/s41598-017-01587-6)

Supplementary Information

Accumulation of multiple neurodegenerative disease-related proteins in familial Frontotemporal Lobar Degeneration associated with granulin mutation

Masato Hosokawa*, Hiromi Kondo, Geidy E Serrano, Thomas G Beach, Andrew C Robinson, David M Mann, Haruhiko Akiyama, Masato Hasegawa, Tetsuaki Arai

Supplementary Figure 1. Evaluation of the degree of protein accumulation.

Representative visual fields in the temporal lobe of the *GRN* mutation cases for – (negative), + (mild), ++ (moderate) and +++ (severe) of tau immunohistochemistry with AT8, as well as of phosphorylated α-synuclein immunohistochemistry with the 1175 antibody. The scale bar in A applies to B-D (100 μm) and in E applies to F-H (200 μm).

Supplementary Figure 2. Photomicrograph of phosphorylated tau immunohistochemistry of the temporal lobe of the *GRN* mutation cases (Study B, paraffin-embedded sections).

AT8-positive structures were observed mainly in the cortex in cases 8 (A), 9 (B), 10 (C) 11 (D), 12 (E), 13 (F), 14 (G) and 16 (I). Case 15 (H) was AT8 negative. Sections were

counterstained with Kernechtrot stain solution. The scale bar in A applies to all photomicrographs (100 µm).

Supplementary Figure 3. Immunoblot analyses with 3R and 4R-tau specific antibodies in sarkosyl-insoluble fraction of *GRN* mutation cases.

Immunoblotting analysis was visualized using RD3 (left) or anti-4R (right) antibody for detecting tau in the sarkosyl-insoluble fraction from cases 3 (lane 1) and 4 (lane 2) with *GRN* mutations, and a case each of CBD (lane 3), PSP (lane 4) and AD (lane 5). Bottom panels were overexposed images. Molecular weight markers are shown on the right (kDa).

Supplementary Figure 4. Immunoblot analyses with anti-phosphorylated α-synuclein antibodies in sarkosyl-insoluble fraction of *GRN* mutation cases.

Immunoblotting analysis was visualized using 1175 (left) or pSyn#64 (right) antibody for detecting α-synuclein in the sarkosyl-insoluble fraction from cases 1 (lane 1), 2 (lane 2), 3 (lane 3) and 4 (lane 4) with *GRN* mutations, and a case of DLB (lane 5). Arrowhead indicates α-synuclein monomer. Molecular weight markers are shown on the right (kDa).

Supplementaly figure 1, Hosokawa et al.

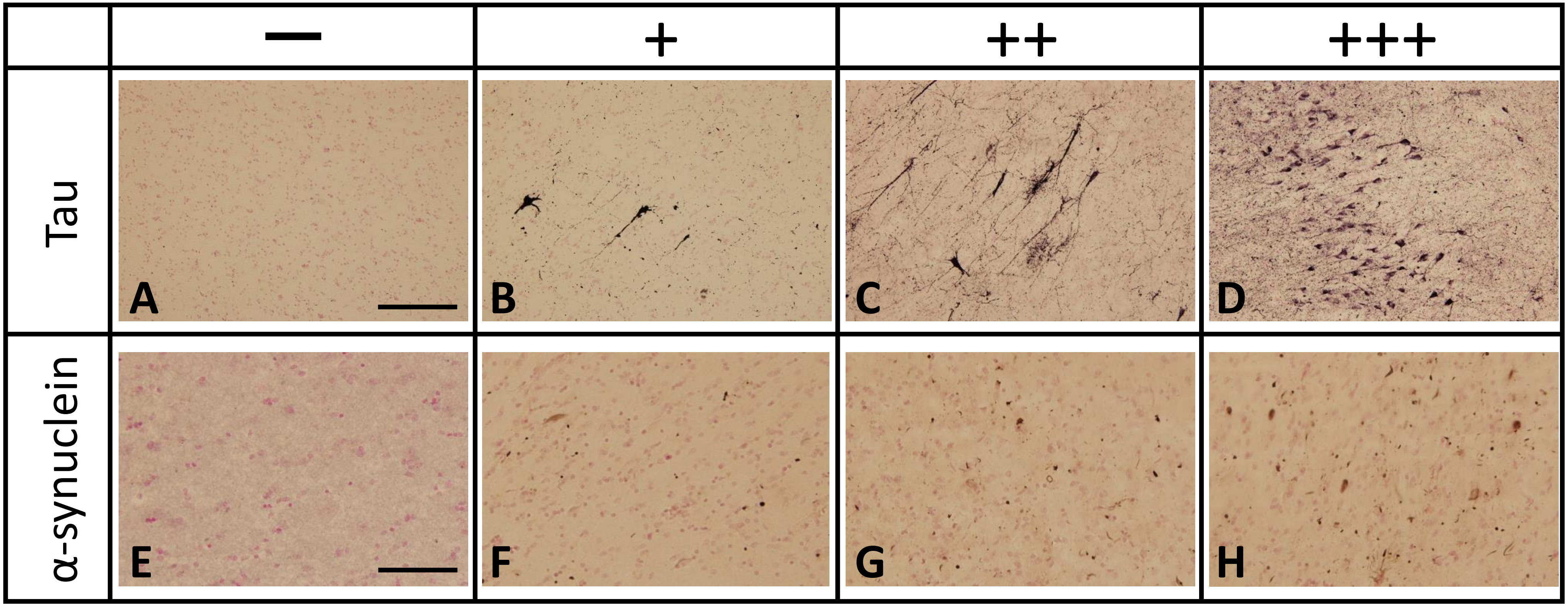

Supplementary Figure 2, Hosokawa et al.

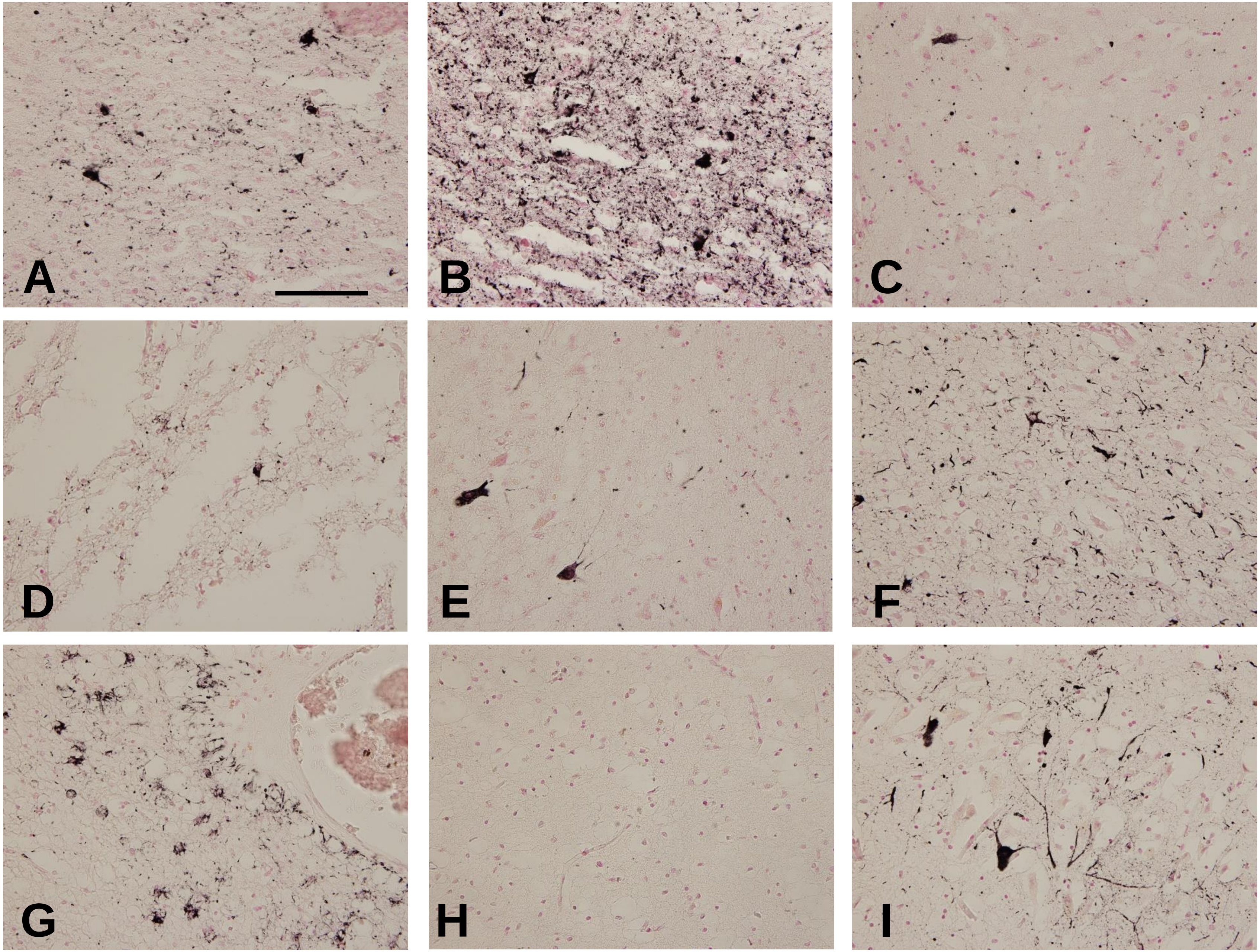

Supplementary Figure 3,
Hosokawa et al.

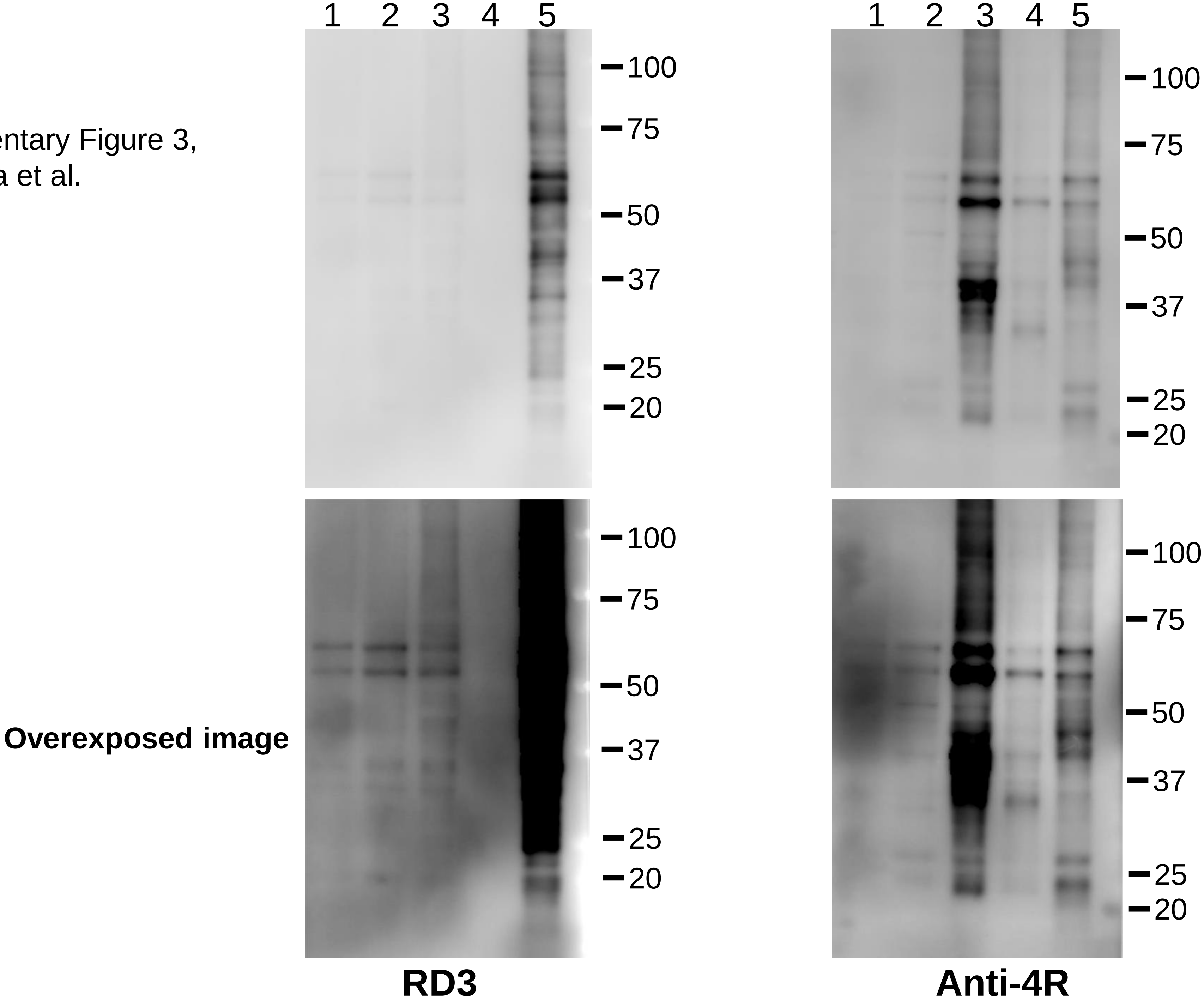

Supplementary Figure 4, Hosokawa et al.

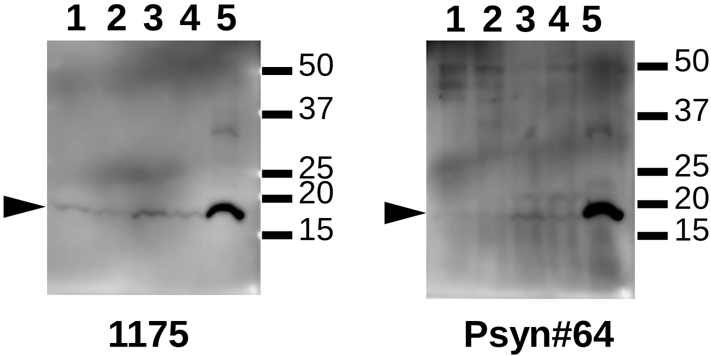

Supplement: Supplementary file 1 — Supplementary Information [file 41598_2017_1587_MOESM1_ESM.pdf]
